# Chemical Constituents from *Hericium erinaceus* Promote Neuronal Survival and Potentiate Neurite Outgrowth via the TrkA/Erk1/2 Pathway

**DOI:** 10.3390/ijms18081659

**Published:** 2017-07-30

**Authors:** Cheng-Chen Zhang, Chen-Yu Cao, Miwa Kubo, Kenichi Harada, Xi-Tao Yan, Yoshiyasu Fukuyama, Jin-Ming Gao

**Affiliations:** 1 Shaanxi Key Labotory of Natural Products & Chemical Biology, College of Chemistry & Pharmacy, Northwest A&F University, Yangling 712100, China; 08cxzk3zc@163.com (C.-C.Z.); caocy@nwsuaf.edu.cn (C.-Y.C.); xty@nwsuaf.edu.cn (X.-T.Y.); 2 Faculty of Pharmaceutical Sciences, Tokushima Bunri University, Tokushima 770-8514, Japan; miwa-k@ph.bunri-u.ac.jp (M.K.); kenichi@ph.bunri-u.ac.jp (K.H.)

**Keywords:** *Hericium erinaceus*, erinacine A, cyathane diterpenoid, 4-chloro-3,5-dimethoxybenzoic methyl ester, PC12 cell, neuritogenesis

## Abstract

*Hericium erinaceus* is a culinary-medicinal mushroom used traditionally in Eastern Asia to improve memory. In this work, we investigated the neuroprotective and neuritogenic effects of the secondary metabolites isolated from the MeOH extract of cultured mycelium of *H. erinaceus* and the primary mechanisms involved. One new dihydropyridine compound (**6**) and one new natural product (**2**) together with five known compounds (**1**,**3**–**5**,**7**) were obtained and their structures were elucidated by spectroscopic analysis, including 2D NMR and HRMS. The cell-based screening for bioactivity showed that 4-chloro-3,5-dimethoxybenzoic methyl ester (**1**) and a cyathane diterpenoid, erincine A (**3**), not only potentiated NGF-induced neurite outgrowth but also protected neuronally-differentiated cells against deprivation of NGF in PC12 pheochromocytoma cells. Additionally, compound 3 induced neuritogenesis in primary rat cortex neurons. Furthermore, our results revealed that TrkA-mediated and Erk1/2-dependant pathways could be involved in **1** and **3**-promoted NGF-induced neurite outgrowth in PC12 cells.

## 1. Introduction

Neurotrophins (NTs), such as nerve growth factor (NGF), have been extensively explored for their essential contribution for the survival and differentiation in neuronal cells [1,2]. There is growing evidence that the edecline of NTs is an important factor in the pathogenesis of neurodegenerative diseases (ND) [3]. In fact, genetic or direct induction of NGF has already been elucidated as a promising treatment for ND [4,5]. However, NGF’s high molecular weight restricts its delivery across the blood-brain barrier (BBB). Hence, NGF-mimic or NGF-potentiated small molecules show significant therapeutic potential for their pharmacological advantages [6,7].

*Hericium erinaceus* (Bull.: Fr) Pers is an edible and medicinal mushroom widely consumed in Asian countries and has been paid more and more attention recently for its prominent neurotrophic and neuroprotective properties [8,9,10,11,12,13]. In in vitro assays, extracts from *H. erinaceus* were reported to stimulate NGF synthesis and/or promote NGF-induced neurite outgrowth in various cell types [14,15]. In in vivo assays, *H. erinaceus’*s fruiting bodies exhibited significant anti-dementia activity [16,17]. The aqueous extract from fruiting bodies also promoted the injured nerve regeneration in a Sprague-Dawley rat model [18].

In fact, the active components obtained from *H. erinaceus*, as well as other mushrooms, can be divided into two groups based on their molecular weight [10]. Erinacines and hericenones are representative small molecules with significant neurotrophic and neuroprotective effects and high convenience to cross the BBB [19,20]. Erinacines A-I showed a stronger ability to increase mRNA expression in NGF synthesis than epinephrine as a positive control in murine astroglial cells [21]. Erinacine P, Q, and their biosynthetically-related compounds were also reported to induce NGF synthesis [21]. In vivo, erinacine A, a cyathane diterpenoid, obviously upregulated the level of NGF in the rat’s locus caeruleus and hippocampus other than the cerebral cortex [22]. Additionally, erinacine A effectively prevented PC12 apoptosis induced by glutamate insult [23]. Erinacine A also protected neurons from death by inhibiting nitrotyrosine-containing proteins, phosphorylation of p38 mitogen-activated protein kinases (MAPK), CCAAT/enhancer-binding protein (C/EBP), and homologous protein (CHOP) in a rat model of global ischemic stroke [24]. Above all, these findings revealed erinacine A as one of the definite elements responsible for the NGF-synthesis stimulating and neuroprotective activities of *H. Erinaceus*, which can be further proved by the fact that erinacine A was able to obviously ameliorate pathologies in Alzheimer’s disease (AD) and Parkinson’s disease (PD) mouse models [25,26]. However, little is known about the direct neuritogenic activity and its detailed mechanism of the functional erinacines, such as erinacine A.

The objective of this research was to explore the potential neuroprotective and neuritogenic effects of 4-chloro-3,5-dimethoxybenzoic methyl ester (**1**) and erinacine A (**3**), components of *H. erinaceus* cultured mycelium extracts, on PC 12 cells as well as primary cultured rat cortex neurons. Furthermore, the involved signaling pathways were revealed by monitoring phosphorylation level as well as cell morphological changes due to selective inhibitors. Our hypothesis is that compounds **1** and **3** can strengthen the activation of tyrosine kinase A (TrkA) triggered by NGF to promote neurite outgrowth by upregulating related downstream signaling, such as extracellular signal-regulated protein kinase 1/2 (Erk1/2) which is essential for neurite outgrowth in PC12 cells.

## 2. Results and Discussion

### 2.1. Structural Elucidation of Compounds ***1**–**7***

**Figure 1:**
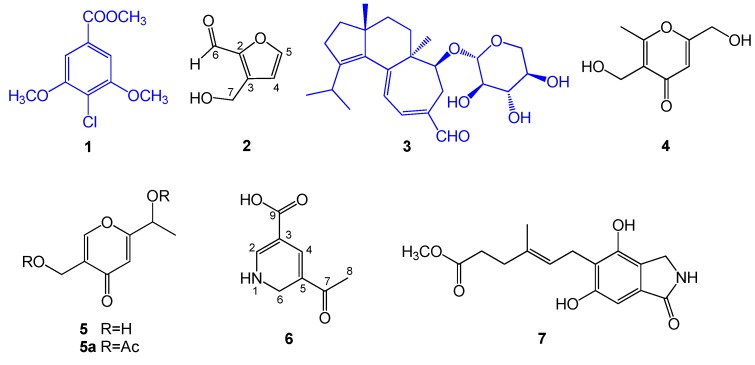
Structures of compounds **1**–**7** from *Hericium erinaceus*.

One new compound (**6**), one new natural product 3-(hydroxymethyl)-2-furaldehyde (**2**) and five known compounds, namely, 4-chloro-3,5-dimethoxybenzoic methyl ester (**1**) [27], erinacine A (**3**) [28], herierin III (**4**) [29], herierin IV (**5**) [29], and erinacerin G (**7**) [30], were isolated from the MeOH extract of the mycelium of H. erinaceus (Figure 1). The known compounds were identified by comparing their spectral data with those reported in the literature.

Compound **6** was isolated as a white, amorphous powder. Its molecular formula was determined to be C_8_H_9_NO_3_ from HRCIMS data at *m*/*z* 168.0658, [M + H]^+^ (calcd for C_8_H_10_NO_3_, 168.0661), with the five degrees of unsaturation (Figure S1). Its IR spectrum displayed absorption bands of hydroxyl and NH (3354 cm^−1^), conjugated C=O (1659 cm^−1^), and conjugated double bond (1626 cm^−1^) (Figure S2). The ^1^H NMR spectrum had resonances corresponding to a methyl proton [*δ*_H_ 2.69 (3H, s)], nitrogenous methylene protons [*δ*_H_ 4.73 (2H, d, *J* = 4.9 Hz)], two olefinic protons [*δ*_H_ 6.88 (1H, d, *J* = 0.5 Hz), 8.90 (1H, s)], one carboxylic proton [*δ*_H_ 12.56 (1H, s)] (Table 1). The ^13^C NMR spectrum (Table 1) had resonances corresponding to a methyl carbon (*δ*_C_ 26.4), a nitrogenous methylene carbon (*δ*_C_ 64.2), four olefinic carbons (*δ*_C_ 109.3, 116.6, 152.1, 166.1), one carboxyl carbonyl carbon (*δ*_C_ 168.3), and one ketone carbonyl carbon (*δ*_C_ 203.8) (Table 1).

**Figure 2:**
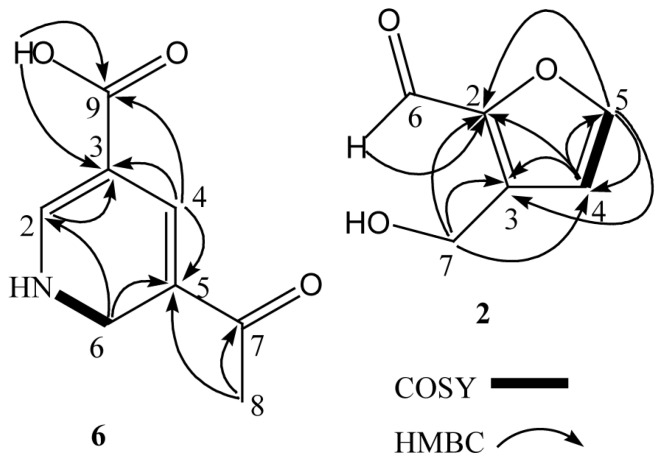
Key ^1^H-^1^H COSY and HMBC correlations of **6** and **2**.

Its COSY correlations between NH/H-6 showed the presence of a fragment –NH–CH_2_– (Figure 2). The presence of a 1,2-dihydropyridine ring was supported by the HMBC correlations from H-2 to C-3, from H-4 to C-3, C-5, and from H-6 to C-2, C-5. The carboxylic group was located at C-3 (*δ*_C_ 116.6) due to HMBC correlations between the carboxylic proton and C-3, and H-4 and C-9 (*δ*_C_ 168.3). The remaining acetyl group was placed at C-5 (*δ*_C_ 166.1), which was supported from the HMBC correlations of H-8 with the quaternary carbon C-5 (Table 1). The aforementioned data allowed us to assign the structure of **6** as 5-acetyl-1,6-dihydro-3-pyridinecarboxylic acid (The related NMR spectra can be seen in Figures S3–S7).

Compound **2** was obtained as yellow oil and its molecular formula was assigned as C_6_H_6_O_3_ by HREIMS. The ^1^H NMR spectrum had resonances corresponding to an oxygenated methylene proton [*δ*_H_ 4.72 (2H, s)], two olefinic protons [*δ_H_* 6.52 (1H, d, *J* = 3.5 Hz), 7.22 (1H, d, *J* = 3.5 Hz)], and an aldehyde proton [*δ*_H_ 9.58 (1H, s)]. The ^13^C NMR spectrum had resonances corresponding to an oxygenated methylene carbon (*δ_C_* 57.8), four olefinic carbons (*δ*_C_ 110.2, 123.0, 152.5, 160.8), and an aldehyde carbon (*δ*_C_ 177.9). Analysis of its ^1^H NMR and COSY correlations between H-4/H-5 (Figure 2) revealed the presence of a 2,3-disubstituted furan ring at *δ*_H_ 6.52 (1H, d, *J* = 3.5 Hz), 7.22 (1H, d, *J* = 3.5 Hz). The HMBC correlations between H-4 (*δ*_H_ 6.52) and C-2, C-3, and C-5, and H-5 (*δ*_H_ 7.22) and C-2, C-3, and C-4 confirmed the presence of the furan moiety. The HMBC correlations from the aldehyde proton H-6 (*δ*_H_ 9.58) to C-2, and from H-7 (*δ*_H_ 4.72) to C-2, C-3, and C-4 (Figure 2) indicated the aldehyde and hydroxymethyl groups to be attached to C-2 and C-3 of the furan ring, respectively. On the basis of the above data, compound **2** was identified as 3-(hydroxymethyl) -2-furaldehyde. This compound was previously converted starting from glycolaldehyde using aluminum chloride as a catalyst [31].

### 2.2. Compounds ***1*** and ***3*** Promoted NGF-Induced Neurite Outgrowth in PC12 Cells

**Figure 3:**
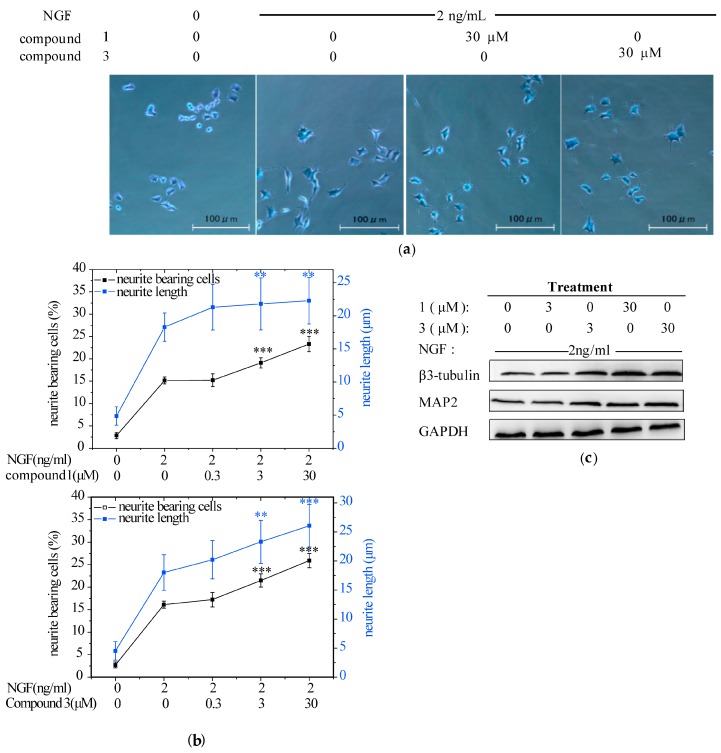
The effect of compounds **1** and **3** from the mycelium of *H. erinaceus* on promoting nerve growth factor (NGF)-induced neurite outgrowth in PC12 cells. PC12 cells were seeded in collagen-coated 24-well plates in normal serum medium for 24 h, then changed to a low-serum medium (2% HS and 1% FBS) and exposure to vehicle (0.1% DMSO) as a negative control, NGF (2 ng/mL) as a positive control, or NGF (2 ng/mL)+1 or 3 (0.3, 3, 30 μM) for additional 96 h. Scale bar: 100 μm. (**a**) Cell morphology was observed and photographed as described in the analysis of neurite outgrowth; (**b**) Neurite bearing cells and neurite length were measured as described in the analysis of neurite outgrowth. Data are expressed as the mean ± SD from three independent experiments. ** *p* < 0.01, *** *p* < 0.001 represent significant differences compared with NGF-treated PC12 cells (positive control) (Analysis of variance (ANOVA) followed by Dunnett’s test); (**c**) Western blotting analysis of the protein expression of neuronal differentiation markers: β3-tubulin and MAP2 (microtubule associated protein 2). Glyceraldehyde-3-phosphate dehydrogenase (GAPDH) was detected as the control of protein loading.

At first, we evaluated the neuritogenic effects of the MeOH extract from the mycelium of *H. erinaceus* by using PC12 cells since it was a widely-employed in vitro model for neuronal differentiation and neurite outgrowth studies. The extract (12.5–200 μg/mL) promoted NGF-induced neurite outgrowth in PC12 cells (Figure S8). Then, PC12 cells were treated with NGF (2 ng/mL) alone or in combination with the seven isolated compounds at the concentrations of 0.3, 3, and 30 μM to identify the specific components responsible for the neurotrophic activity. Consequently, **1** and **3** enhanced the growth of neurites in a dose-dependent manner and significantly increased the maximal neurite length in the presence of NGF (Figure 3a,b). In parallel, the NGF-induced expression of neuronal differentiation marker proteins (i.e., MAP2 and β3-tubulin), were also obviously upregulated by the combination of **1** or **3** with NGF (Figure 3c). Taken together, these data indicated that compounds **1** and **3** could effectively induce neuritogenesis when the PC12 cells were triggered by a low concentration of NGF. This finding agrees well with other research. Hericenones C-E from *H. Erinaceus* markedly potentiated the neurite outgrowth of PC12 cells in the presence of NGF (5 ng/mL) [32]. Treatment of PC12 cells with corallocins A- or C-conditioned media produced by 1321N1 astrocytes resulted in significant increase of neurite outgrowth [33]. Furthermore, it was demonstrated that increasing neurite outgrowth activity in PC12 cells by the above compounds exhibited a strong correlation with the stimulation of NGF synthesis [32,33]. However, erinacine A was unable to stimulate NGF synthesis with or without NGF in PC12 cells (Figure S9). This indicated that erinacine A induced neuritogenesis in PC12 acting as a stimuli for NGF potency instead of NGF level.

### 2.3. Compounds ***1*** and ***3*** Promoted NGF-Induced Neurite Outgrowth by Activating TrkA/Erk1/2 Pathway in PC12 Cells

It was reported that stimulation of PC12 cells with NGF induces TrkA phosphorylation. Tyr-490-phophorylated form TrkA is required for Shc association and activation of the Ras-MAP kinase cascade, which is one of vital pathways involved into NGF-induced neurite outgrowth [34,35,36]. To explore the possible mechanism underlying the neurotrophic activity, the influences of compound **1** and **3** on TrkA and Erk1/2 were examined.

#### 2.3.1. Specific TrkA and Erk1/2 Inhibitors Reduced Compounds **1** and **3**-Potentiated Neurite Outgrowth in PC12 Cells

**Figure 4:**
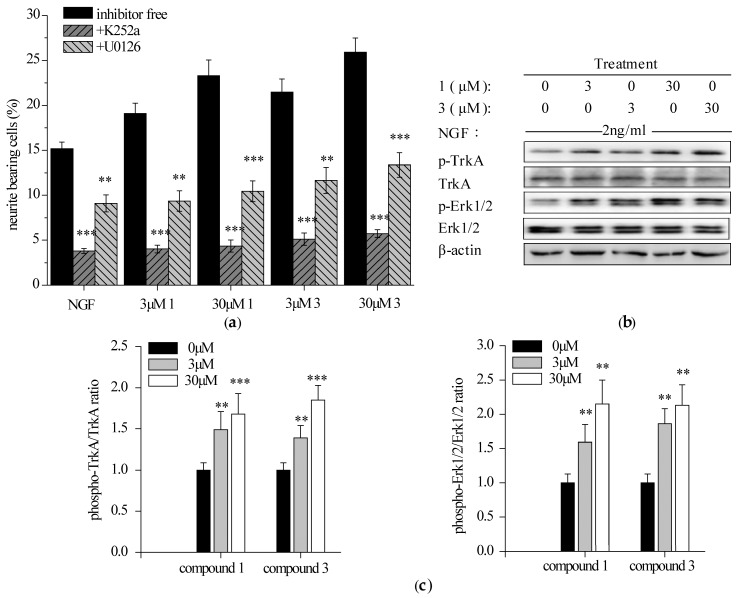
Effects of TrkA and Erk1/2 inhibitors on the NGF-induced neurite outgrowth and results of TrkA (tyrosine kinase A) and Erk1/2 (extrallular signal-regulated protein kinase 1/2) phosphorylation in PC12 cells in the presence of **1** and **3**. (**a**) Values show the percentage of neurite-bearing cells after 1 h pretreatment with or without inhibitors followed by 96 h exposure to 2 ng/mL NGF or indicated agents (with 2 ng/mL NGF). Data are expressed as the mean ± SD from three independent experiments. ** *p* < 0.01, *** *p* < 0.001 represent significant differences compared with the inhibitor-free groups (ANOVA followed by Dunnett’s test); (**b**) Western blotting analysis of **1** and **3** on the NGF-induced TrkA and Erk1/2 phosphorylation in PC12 cells. The cells were treated with NGF (2 ng/mL) and compound **1** or **3** (3 and 30 μM), alone or in combination for 15 min (TrkA) or 30 min (Erk1/2). β-actin was detected as the control of protein loading; (**c**) Densitometric analysis of protein phosphorylation levels on Western blotting (**b**). Normalized intensity of phospho-protein versus protein is expressed as the mean ± SD from three independent experiments. ** *p* < 0.01, *** *p* < 0.001 represent significant differences compared with NGF-treated PC12 cells (positive control) (ANOVA followed by Dunnett’s test).

Selective TrkA inhibitor (k252a, 200 nM) and Erk1/2 inhibitor (U0126, 50 nM), were added for 1 h prior to the addition of NGF and **1** or **3**, alone or in combination for 96 h. Both antagonism of TrkA and ERK1/2 markedly reduced the percentage of neurite-bearing cells (Figure 4a). Specifically, k252a abolished the neuritogenesis induced by NGF and **1** or **3**, alone or in combination at a level comparable to the DMSO control (around 5%). Similarly, the neuritogenic effect of iridoidcatalpol from *Rehmannia glutinosa* (Shengdihuang) was also completely abolished by k252a in a primary culture of forebrain neurons [37]. In contrast, the activity of potentiating NGF-mediated PC12 neurite outgrowth by hericenone E was found to be partially blocked by k252a [32]. Furthermore, U0126 reduced compounds **1** and **3**-potentiated neurite outgrowth by 50%. It was consistent with the fact that Erk1/2 was not the sole pathway involved into the NGF-induced neurite outgrowth in PC12 cells [36]. Taken together, it indicated that NGF-induced neurite outgrowth promoted by compounds **1** and **3** may be entirely TrkA-dependent and partially Erk1/2-mediated.

#### 2.3.2. Compounds **1** and **3** Enhanced NGF-Induced TrkA and Erk1/2 Phosphorylation in PC12 Cells

PC12 cells were starved and exposed to NGF and **1** or **3**, alone or in combination for 15 min (TrkA) or 30 min (Erk1/2). The phosphorylation of TrkA (Y490) and a downstream protein, Erk1/2, were analyzed by Western blotting. Both **1** and **3** promoted NGF-induced phosphorylation of TrkA (Y490) and Erk1/2 in a concentration-dependent manner (Figure 4b,c). Previous reports have revealed that upregulation of TrkA and/or Erk1/2 phosphorylation by some natural products agreed well with their potency to promote neurite outgrowth [32,38,39]. It is, thus, predicted that compounds **1** and **3** may mediate the interaction between NGF and its receptor TrkA, enhance the activation of TrkA and its downstream signaling Erk1/2 and finally potentiate NGF-induced neurite outgrowth in PC12 cells.

### 2.4. Compounds ***1*** and ***3*** Prevented the Death of Neuronally-Differentiated PC12 Cells against the Removal of NGF

**Figure 5:**
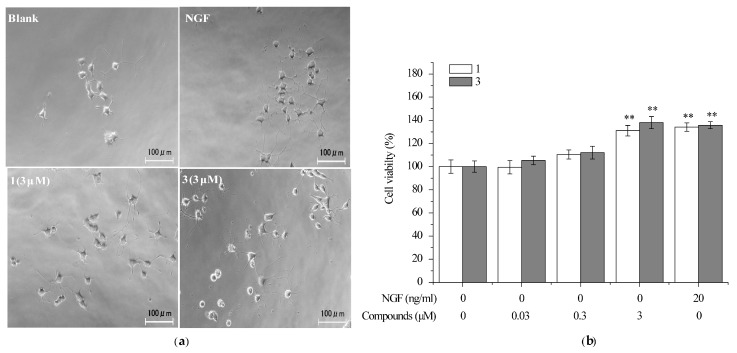
The effect of compounds **1** and **3** on preventing the death of neuronally-differentiated PC12 cells after removal of NGF. PC12 cells were seeded in collagen-coated 96-well plates in normal serum medium for 24 h, then changed to low serum medium (2% HS and 1% FBS) containing NGF (30 ng/mL). After seven days, PC12 cells were exposure to vehicle (0.1% DMSO) as a negative control, NGF (20 ng/mL) as a positive control, or 1, 3 (0.03, 0.3, 3 μM) for an additional 48 h. Scale bar: 100 μm. (**a**) Representive cell morphology images and (**b**) cell viability was assessed by MTT assay. Data are expressed as the mean ± SD from three independent experiments. ** *p* < 0.01 represent significant differences compared with vehicle (ANOVA followed by Dunnett’s test).

Differentiated PC12 cells need NGF to maintain survival, proliferation and the phenotypic properties of sympathetic neurons. It was reported that several natural products such as senegenin can protect neurites of differentiated PC12 cells from the removal of NGF [40]. In order to investigate the effect of compounds **1** and **3** on the neuronally-differentiated PC12 without NGF, the cell viability was measured by using MTT assay. PC12 cells were neuronally-differentiated treated with NGF (30 ng/mL) for 7 d. The cells were treated with **1** and **3** at the concentrations of 0.03, 0.3, and 3 μM after NGF withdrawal and cell viability was measured 48 h later. After NGF withdrawal, 50–60% of neuronally-differentiated PC12 cells were dead 48 h later (Figure 5a). At the concentration of 3 μM, the cells treated with **1** and **3** increased NGF-deprived PC12 cell viability up to 131% and 138%, respectively, versus untreated cells, which is comparable with the cells treated with 20 ng/mL NGF (Figure 5b). These results indicated that compounds **1** and **3** were able to protect differentiated PC 12 cells from the removal of NGF. Since compound **1** was reported to protect mouse neuroblastoma N2a (Neuro-2a) cells against endoplasmic reticulum stress [27], our findings confirmed the neuroprtective function of 4-chloro-3,5-dimethoxybenzoic methyl ester.

### 2.5. Compound ***3*** Induced Neurite Outgrowth in Primary Rat Cortex Neurons

**Figure 6:**
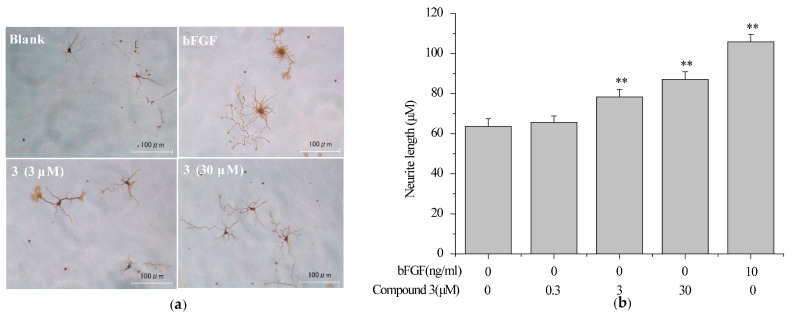
The effect of compound **3** on promoting neurite outgrowth in primary cultured rat cortex neurons. Primary rat cortex neurons were seeded in poly-l-lysine-coated 24-well plates in DMEM for 24 h, then changed to neurobasal medium (2% B27 and 0.5 mM l-glutamine) and exposure to vehicle (0.1% DMSO) as a negative control, bFGF (10 ng/mL) as a positive control, or **3** (0.3, 3, 30 μM) for an additional six days. Scale bar: 100 μm. (**a**) Cell morphology was observed and photographed as described in Section 3.8; (**b**) Neurite length was measured as described in Section 3.8. Data are expressed as the mean ± SD from three independent experiments. ** *p* < 0.01 represent significant differences compared with vehicle (ANOVA followed by Dunnett’s test).

Primary cultured neurons possess majority of the in vivo neuronal properties. Thus, rat cortical neurons were chosen to screen neurotrophic molecules from *H. erinaceum*. As a result, compound **3** was investigated to induce the neurite extension (Figure 6a). The maximal neurite length for the neurons treated with **3** (3 and 30 μM) were significantly larger than untreated neurons, but smaller than the basic fibroblast growth factor (bFGF, 10 ng/mL) control (Figure 6b). These data indicated that compound **3** can mimic NTs finitely in primary rat cortex neurons.

## 3. Materials and Methods

### 3.1. General Experimental Procedures

IR spectra were recorded on a JASCO FT-IR 410 infrared spectrophotometer (JASCO Inc.: Easton, MD, USA). The NMR experiments were performed on a Varian Unity 600 or 500 instrument (Varian Inc.: Palo Alto, CA, USA). Deuterated solvent was used as references for the ^1^H and ^13^C NMR spectra. HREIMS and HRCIMS were performed on an MStation JMS-700 or a JMX-AX 500 spectrometer (JEOL Ltd.: Tokyo, Japan). TLC was carried out with silica gel 60 F254 and PR-18 F254 plates. HPLC was performed on a JASCO PU-1580 pump (JASCO Inc.: Easton, MD, USA) equipped with a JASCO UV-1575 detector (JASCO Inc.: Easton, MD, USA). All solvents used for extraction and isolation were of analytical grade.

### 3.2. Fungal Materials

The fungal strain *H. erinaceum* H6 (accession no. DQ185914) was purchased from the Applied Research Institute of Microbiology, Academy of Agricultural Sciences, Xinjiang, China. It was identified based on 16S rDNA sequence analysis with 99% similarity by Beijing Sunbiotech Co. and has been deposited at Shaanxi Key Labotory of Natural Products and Chemical Biology, Northwest A and F University. *H. erinaceus* was cultured on slants of potato dextrose agar at 25 °C for 10 days. Agar plugs were inoculated in a 500 mL Erlenmeyer flask containing 120 mL of the culture medium and incubated at 25 °C on a rotary shaker at 100 rpm for 10 days. The components of the culture medium were listed as follows: glucose 0.4%, malt extract 1%, yeast extract 0.4% in distilled water. The culture medium was adjusted to pH 6.5 before sterilization. The scale-up fermentation was carried out in 40 × 500 mL Erlenmeyer flasks each containing 80 g of rice and 120 mL of distilled water. Each flask was inoculated with 5.0 mL of the culture medium and incubated at 25 °C for 40 days.

### 3.3. Extraction and Isolation

*H. erinaceus* mycelium was extracted and isolated as previously reported with slight modifications [41]. Briefly the MeOH extract (45 g) was suspended in water and then partitioned with EtOAc and n-BuOH. The EtOAc fraction (4.41 g) was subjected to silica gel column chromatography (CC) using CHCl_3_–MeOH (20:1, 9:1, 7:3, 5:5) to give four fractions (E1–E4).

Fraction E1 (116 mg) was separated by silica gel CC eluted with hexane-EtOAc (4:1, 2:1, 1:1) to give five fractions (E11–E15). Fraction E11 was separated by ODS column chromatography (CC) using 90% MeOH in water and then purified by preparative TLC using hexane-EtOAc (5:1) to obtain compound **1** (8 mg). Fraction E2 (982 mg) was subjected to silica gel CC eluted with CHCl_3_-EtOAc (2:1, 1:1) to give ten fractions (E21–E30). Compound **2** (29.6 mg) was obtained from fraction E24 (80 mg) by silica gel CC using CHCl_3_-EtOAc (9:1). Fraction E29 (82 mg) was separated by ODS CC using MeOH-H_2_O (3:7, 5:5, 7:3) and then purified by RP-HPLC using MeOH-H_2_O (9:1) to obtain **3** (6 mg). Fraction E3 (1.215 g) was separated by silica gel CC eluted with CHCl_3_-MeOH-H_2_O (7:3:0.5) to give two fractions (E31, E32). Fraction E31 (956 mg) was separated by silica gel CC using hexane-EtOAc (1:4, 0:1) to afford four fractions (E311–E314). Fraction 314 (276 mg) was separated on Sephadex LH-20 CC eluted with MeOH to give three fractions (E3141–E3143). Fraction 3142 (71 mg) was further separated by ODS CC eluted with MeOH-H_2_O (1:2) to give three fractions (E31421–E31423). Compound **4** (5 mg) was obtained from fraction E31421 (32 mg) by RP-HPLC using 5% acetonitrile in water. Compound **5** (10 mg) was obtained from fraction E32 (215 mg) by RP-HPLC using MeOH-H_2_O (4:6). Fraction E4 (1.08 g) was separated by ODS CC using MeOH-H_2_O (5:5, 7:3) to give three fractions (E41–E43). Fraction E41 (459 mg) was separated by silica gel CC eluted with EtOAc-MeOH (9:1) to give two fractions (E411, E412). Fraction E412 (112.2 mg) was separated by silica gel CC using CHCl_3_-MeOH (9:1) and then purified by RP-HPLC using 10% MeOH in water to obtain **6** (1 mg). Fraction E42 (384 mg) was separated by silica gel CC using a gradient of CHCl_3_-MeOH (20:1, 10:1) to afford four fractions (E421–E424). Fraction 421 (116.3 mg) was further separated by ODS CC using MeOH-H_2_O (1:1) to give three fractions (E4211–E4213). Compound **7** (2 mg) was obtained from fraction E4213 (24.8 mg) by silica gel CC eluted with CHCl_3_-EtOAc (1:5).

Compound **2** (=3-(hydroxymethyl)-2-furaldehyde):yellow oil; ^1^H NMR (CD_3_OD, 500 M Hz) δ 4.72 (2H, s, H-7), 6.52 (1H, d, *J* = 3.5 Hz, H-4), 7.22 (1H, d, *J* = 3.5 Hz, H-5),9.58 (1H, s, H-6); ^13^C NMR (CD_3_OD, 125 M Hz) δ 57.8 (C-7), 110.2 (C-4), 123.0 (C-5), 152.5 (C-2), 160.8 (C-3), 177.9 (C-6); EIMS (rel int) *m*/*z* 126[M]^+^ (100); HREIMS *m*/*z* 126.0315 [M]^+^ (calcd for C_6_H_6_O_3_, 126.0317).

Acetylation of compound **5**. To a solution of **5** (3 mg) in pyridine (1 mL) was added acetic anhydride (1 mL), and the mixture stood at RT for 24 h. The reaction mixture was concentrated to dryness, the residue were purified on silica gel CC using EtOAc, affording a new diacetate 5a (2.1 mg) as an off-white gum. ^1^H NMR (CDCl_3_, 500 M Hz) δ 1.50 (3H, d, *J* = 6.0 Hz), 2.10 (3H, d, *J* = 3.0 Hz), 2.16 (3H, d, *J* = 1.5 Hz), 4.90 (2H, s), 5.90 (1H, dd, *J* = 6.5, 1 Hz), 6.36 (1H, s), 7.79 (1H, d, *J* = 0.5 Hz); ^13^CNMR (CDCl_3_, 125 M Hz) δ 19.9, 20.6, 21.2, 61.1, 65.7, 114.7, 129.6, 151.9, 162.2, 169.6, 169.9, 176.8; HRCIMS *m*/*z* 255.0874 [M + H]^+^(calcd for C_12_H_15_O_6_, 255.0863).

Compound **6**: amorphous, white powder; IR (film) ν_max_ 3354 (OH, NH), 1659 (C=O), 1626, 1541, 1507, 1370, 1215, 1067 cm^−1^; ^1^H NMR (CDCl_3_, 600 M Hz) and ^13^C NMR (CDCl_3_, 150 M Hz) data, Table 1; CIMS *m*/*z* 168 [M + H]^+^; HRCIMS *m*/*z* 168.0658 [M + H]^+^ (calcd for C8H10NO3, 168.0655).

### 3.4. Cell Culture

PC12 cells were provided from Professor Yoshiyasu Fukuyama’s laboratory and were grown in high-glucose Dulbecco’s Modified Eagle Medium (DMEM) with 10% heat-inactivated horse serum (Gibco BRL Co.Ltd.: Gaithersburg, MD, USA), 5% heat-inactivated fetal bovine serum (Gibco BRL), 1% penicillin/streptomycin. The cells were cultivated in an atmosphere with 5% CO_2_ at 37 °C.

Primary rat cortex neurons were purchased from Gibco BRL. The cells were seeded into poly-l-lysine (Sigma-Aldrich Inc.: Saint Louis, MI, USA) coated 24-well plates at a density of 2 × 10^4^ cells/mL. After 24 h, the medium was replaced with Neurobasal medium supplement with B27 (2%), l-glutamine (0.5 mM) and 1% penicillin/streptomycin. The cells were maintained in a humidified 5% CO_2_ incubator.

### 3.5. MTT Assay for Cell Viability

PC12 cells were seeded in collagen coated 96-well plates at a density of 8 × 10^3^ cells/mL. After 24 h, the culture medium was replaced with the differential medium containing 30 ng/mL of NGF for seven days. After NGF withdrawal, the cells were treated with compounds for 48 h. Then the cells were incubated with of MTT (0.5 mg/mL) for 2 h at 37 °C. The medium was carefully removed and 100 μL of 50% DMSO/50% EtOH was added into each well. The absorbance was measured at 560 nm by a microplate reader. The results were expressed as the percentage of MTT reduction, assuming the absorbance of blank control was 100%.

### 3.6. Assay of Neurite Outgrowth of PC12 Cells

The neurite outgrowth was measured as previously reported [42,43]. Briefly PC12 cells were seeded in poly-l-lysine-coated 24-well plates at a density of 8 × 10^3^ cells/mL with normal serum medium for 24 h, and then treated with the drugs and NGF, alone or in combination. PC12 cells were photographed by an inverted microscope and examined for the percentage of neurite-bearing cells relative to the total cells in the field or the average of maximal neurite length in individual cells.

### 3.7. Western Blotting Analysis

PC12 cells (1 × 10^6^ cells/mL) were seeded in poly-l-lysine-coated six-well plates in normal serum medium for 24 h, then shifted to low serum medium for 14 h prior to exposure to vehicle (0.1% DMSO) or indicated reagents. Cells were lysed on ice for 30 min in RIPA buffer, 10 mM PMSF (Beyotime Institute of Biotechnology, Shanghai, China), 1% protein inhibitor cocktail (Sigma-Aldrich China Inc.: Shanghai, China). Lysates were centrifuged at 14,000 rpm for 15 min at 4 °C. The protein concentration was measured by the BCA protein assay kit (Solarbio Science and Technology Co., Ltd., Beijing, China). Each sample (30 μg) was separated by SDS-PAGE in 8–12% gels, transferred to a PVDF membrane (0.45 μm, Solarbio Science and Technology Co., Ltd.: Beijing, China). The membranes were blocked at room temperature in 5% BSA in TBST (20 mM Tris-HCl, 150 mM NaCl, 0.1% Tween 20 (Polysorbate 20)) for 1 h. Blots were incubated with the appropriate primary antibodies (Cell Signaling Technology, Inc., Beverly, MA) overnight at 4 °C, detected with HRP-conjugated secondary antibodies (Beyotime Institute of Biotechnology, Shanghai, China) for 1 h at room temperature and finally visualized by ECL chemiluminescence reagents (Beyotime Institute of Biotechnology: Shanghai, China).

### 3.8. Immunocytochemical Staining

Primary rat cortex neurons were seeded onto poly-l-lysine-coated 24-well plate for 24 h and treated with compounds in complete neurobasal medium. After six days, the cells were fixed with 4% paraformaldehyde for 20 min, blocked in 0.3% H_2_O_2_ for 20 min, permeabilized with 0.1% Triton X-100 for 20 min. The cells were then incubated with anti-MAP2 antibody in PBS at 4 °C overnight. The bound antibodies were detected by secondary antibody for 60 min at room temperature. The cells was stained using 3,3’-daminobenzidine (DAB) and photographed by an inverted microscope and examined by measuring the length of the longest neurite in each cell. The cells treated with bFGF (10 ng/mL) served as a positive control.

### 3.9. Statistical Analysis

Data were expressed as mean ± standard error (SD). All experiments were carried out at least three times. The results were analyzed by ANOVA followed by Dunnett’s test, with *p* < 0.01 and *p* < 0.001 taken to indicate significant difference.

## 4. Conclusions

In the present study, one new compound 6 and one new natural product 2 from the cultured mycelium of *H. erinaceus* were elucidated. Two known compounds, 1 and 3, have been explored with new neurotrophic and neuroprotective activities. Specifically, compounds 1 and 3 can effectively protect the neuronally-differentiated PC12 cells against deprival of NGF. Compound 3 can mimic finitely neuritogenic activity of NTs in primary rat cortex neurons. Compounds 1 and 3 can also obviously potentiate NGF-induced neurite outgrowth independent of the stimulation of NGF synthesis in PC12 cells. Furthermore, analysis of cellular signaling pathways revealed that NGF-induced neurite outgrowth potentiated by compounds 1 and 3 is completely TrkA-mediated and partially Erk1/2-dependent. Collectively, these facts strengthen the perception that *Hericium erinaceus* as a potential agent to reduce the risk of various neurodegenerative diseases, which may offer useful benefits to help the development of new drugs against these diseases.

## Figures and Tables

**Table 1:** ^1^H (600 M Hz, CDCl_3_) and ^13^C NMR data (150 M Hz, CDCl_3_) of **6**.

Position	*δ*_H_	*δ*_C_	HMBC
2	6.88 (1H, d, *J* = 0.5 Hz)	109.3	C-3
3		116.6	
4	8.90 (1H, s)	152.1	C-3, C-5, C-9
5		166.1	
6	4.73 (2H, d, *J* = 4.9 Hz)	64.2	C-2, C-5, C-8
7		203.8	
8	2.69 (3H, s)	26.4	C-5, C-7
9		168.3	
–NH	3.33 (1H, m)		
–COOH	12.56 (1H, s)		C-3, C-9

## Supplementary Materials

The following are available online at www.mdpi.com/1422-0067/18/8/1659/s1.

## References

[1] Kaplan D.R., Stephens R.M. (1994). Neurotrophin signal transduction by the Trk receptor. J. Neurobiol..

[2] Akagi M., Matsui N., Akae H., Hirashima N., Fukuishi N., Fukuyama Y., Akagi R. (2015). Nonpeptide neurotrophic agents useful in the treatment of neurodegenerative diseases such as Alzheimer’s disease. J. Pharmacol. Sci..

[3] Tiwari S.K., Chaturvedi R.K. (2014). Peptide therapeutics in neurodegenerative disorders. Curr. Med. Chem..

[4] Allen S.J., Watson J.J., Shoemark D.K., Barua N.U., Patel N.K. (2013). GDNF, NGF and BDNF as therapeutic options for neurodegeneration. Pharmacol. Ther..

[5] Xu C.J., Wang J.L., Jin W.L. (2016). The emerging therapeutic role of NGF in Alzheimer’s disease. Neurochem. Res..

[6] Cheng X., Harzdorf N., Khaing Z., Kang D., Camelio A.M., Shaw T., Schmidt C.E., Siegel D. (2012). Neuronal growth promoting sesquiterpene-neolignans; syntheses and biological studies. Org. Biomol. Chem..

[7] Shi X.W., Liu L., Gao J.M., Zhang A.L. (2011). Cyathane diterpenes from Chinese mushroom Sarcodon scabrosus and their neurite outgrowth-promoting activity. Eur. J. Med. Chem..

[8] Gao J.M. (2006). New biologically active metabolites from Chinese higher fungi. Curr. Org. Chem..

[9] Lu Q.Q., Tian J.M., Wei J., Gao J.M. (2014). Bioactive metabolites from the mycelia of the basidiomycete *Hericium erinaceum*. Nat. Prod. Res..

[10] Thongbai B., Rapior S., Hyde K.D., Wittstein K., Stadler M. (2015). *Hericium erinaceus*, an amazing medicinal mushroom. Mycol. Prog..

[11] Friedman M. (2015). Chemistry, nutrition, and health-promoting properties of *Hericium erinaceus* (Lion's Mane) mushroom fruiting bodies and mycelia and their bioactive compounds. J. Agric. Food Chem..

[12] Zhang C.C., Yin X., Cao C.Y., Wei J., Zhang Q., Gao J.M. (2015). Chemical constituents from *Hericium erinaceus* and their ability to stimulate NGF-mediated neurite outgrowth on PC12 cells. Bioorg. Med. Chem. Lett..

[13] Phan C.W., David P., Naidu M., Wong K.H., Sabaratnam V. (2015). Therapeutic potential of culinary-medicinal mushrooms for the management of neurodegenerative diseases: Diversity, metabolite, and mechanism. Crit. Rev. Biotechnol..

[14] Mori K., Obara Y., Hirota M., Azumi Y., Kinugasa S., Inatomi S., Nakahata N. (2008). Nerve growth factor-inducing activity of *Hericium erinaceus* in 1321N1 human astrocytoma cells. Biol. Pharm. Bull..

[15] Lai P.L., Naidu M., Sabaratnam V., Wong K.H., David R.P., Kuppusamy U.R., Abdullah N., Malek S.N.A. (2013). Neurotrophic properties of the Lion’s mane medicinal mushroom, *Hericium erinaceus* (Higher Basidiomycetes) from Malaysia. Int. J. Med. Mushrooms.

[16] Mori K., Inatomi S., Ouchi K., Azumi Y., Tuchida T. (2009). Improving effects of the mushroom Yamabushitake (*Hericium erinaceus*) on mild cognitive impairment: A double-blind placebo-controlled clinical trial. Phytother. Res..

[17] Mori K., Obara Y., Moriya T., Inatomi S., Nakahata N. (2011). Effects of *Hericium erinaceus* on amyloid beta(25–35) peptide-induced learning and memory deficits in mice. Biomed. Res..

[18] Wong K.H., Naidu M., David R.P., Abdulla M.A., Abdullah N., Kuppusamy U.R., Sabaratnam V. (2009). Functional recovery enhancement following injury to rodent peroneal nerve by Lion’s Mane mushroom, *Hericium erinaceus* (Bull.: Fr.) Pers. (Aphyllophoromycetideae). Int. J. Med. Mushrooms.

[19] Kawagishi H., Zhuang C. (2008). Compounds for dementia from *Hericium erinaceum*. Drugs Future.

[20] Tang H.Y., Yin X., Zhang C.C., Jia Q., Gao J.M. (2015). Structure diversity, synthesis, and biological activity of cyathane diterpenoids in higher fungi. Curr. Med. Chem..

[21] Ma B.J., Shen J.W., Yu H.Y., Ruan Y., Wu T.T., Zhao X. (2010). Hericenones and erinacines: Stimulators of nerve growth factor (NGF) biosynthesis in *Hericium erinaceus*. Mycology.

[22] Shimbo M., Kawagishi H., Yokogoshi H. (2005). Erinacine A increases catecholamine and nerve growth factor content in the central nervous system of rats. Nutr. Res..

[23] Chang C.H., Chen Y., Yew X.X., Chen H.X., Kim J.X., Chang C.C., Peng C.C., Peng R.Y. (2016). Improvement of erinacine A productivity in *Hericium erinaceus* mycelia and its neuroprotective bioactivity against the glutamate-insulted apoptosis. LWT Food Sci. Technol..

[24] Lee K.F., Chen J.H., Teng C.C., Shen C.H., Hsieh M.C., Lu C.C., Lee K.C., Lee L.Y., Chen W.P., Chen C.C. (2014). Protective effects of *Hericium erinaceus* mycelium and its isolated erinacine A against ischemia-injury-induced neuronal cell death via the inhibition of iNOS/p38 MAPK and nitrotyrosine. Int. J. Mol. Sci..

[25] Tsai-Teng T., Chin-Chu C., Li-Ya L., Wan-Ping C., Chung-Kuang L., Chien-Chang S., Chi-Ying H.F., Chien-Chih C., Shiao Y.J. (2016). Erinacine A-enriched *Hericium erinaceus* mycelium ameliorates Alzheimer’s disease-related pathologies in APPswe/PS1dE9 transgenic mice. J. Biomed. Sci..

[26] Kuo H.C., Lu C.C., Shen C.H., Tung S.Y., Hsieh M.C., Lee K.C., Lee L.Y., Chen C.C., Teng C.C., Huang W.S. (2016). *Hericium erinaceus* mycelium and its isolated erinacine A protection from MPTP-induced neurotoxicity through the ER stress, triggering an apoptosis cascade. J. Transl. Med..

[27] Ueda K., Kodani S., Kubo M., Masuno K., Sekiya A., Nagai K., Kawagishi H. (2009). Endoplasmic reticulum (ER) stress-suppressive compounds from scrap cultivation beds of the mushroom *Hericium erinaceum*. Biosci. Biotechnol. Biochem..

[28] Kawagishi H., Shimada A., Shirai R., Okamoto K., Ojima F., Sakamoto H., Ishiguro Y., Furukawa S. (1994). Erinacines A, B and C, strong stimulators of nerve growth factor (NGF)-synthesis, from the mycelia of *Hericium erinaceum*. Tetrahedron Lett..

[29] Qian F.G., Xu G.Y., Du S.J., Li M.H. (1990). Isolation and identification of two new pyrone compounds from the culture of *Herictum erinaceus*. Yao Xue Xue Bao.

[30] Wang K., Bao L., Qi Q., Zhao F., Ma K., Pei Y., Liu H. (2015). Erinacerins C-L, isoindolin-1-ones with α-glucosidase inhibitory activity from cultures of the medicinal mushroom *Hericium erinaceus*. J. Nat. Prod..

[31] Schwiderski M., Kruse A. (2015). Catalytic effect of aluminium chloride on the example of the conversion of sugar model compounds. J. Mol. Catal..

[32] Phan C.W., Lee G.S., Hong S.L., Wong Y.T., Brkljača R., Urban S., Abd Malek S.N., Sabaratnam V. (2014). *Hericium erinaceus* (Bull.: Fr) Pers. cultivated under tropical conditions: Isolation of hericenones and demonstration of NGF-mediated neurite outgrowth in PC12 cells via MEK/ERK and PI3K-Akt signaling pathways. Food Funct..

[33] Wittstein K., Rascher M., Rupcic Z., Löwen E., Winter B., Köster R.W., Stadler M. (2016). Corallocins A-C, nerve growth and brain-derived neurotrophic factor inducing metabolites from the mushroom *Hericium coralloides*. J. Nat. Prod..

[34] Lange-Carter C.A., Johnson G.L. (1994). Ras-dependent growth factor regulation of MEK kinase in PC12 cells. Science.

[35] Chao M.V., Hempstead B.L. (1995). p75 and Trk: A two-receptor system. Trends Neurosci..

[36] Vaudry D., Stork P.J., Lazarovici P., Eiden L.E. (2002). Signaling pathways for PC12 cell differentiation: Making the right connections. Science.

[37] Wang Z., Liu Q., Zhang R., Liu S., Xia Z., Hu Y. (2009). Catalpol ameliorates beta amyloid-induced degeneration of cholinergic neurons by elevating brain-derived neurotrophic factors. Neuroscience.

[38] Cheng L.H., Ye Y., Xiang L., Osada H., Qi J.H. (2017). Lindersin B from Lindernia crustacea induces neuritogenesis by activation of tyrosine kinase A/phosphatidylinositol 3 kinase/extracellular signal-regulated kinase signaling pathway. Phytomedicine.

[39] Zhao J., Cheng Y.Y., Fan W., Yang C.B., Ye S.F., Cui W., Wei W., Lao L.X., Cai J., Han Y.F. (2015). Botanical drug puerarin coordinates with nerve growth factor in the regulation of neuronal survival and neuritogenesis via activating ERK1/2 and PI3K/Akt signaling pathways in the neurite extension process. CNS Neurosci. Ther..

[40] Jesky R., Chen H. (2016). The neuritogenic and neuroprotective potential of senegenin against Aβ-induced neurotoxicity in PC 12 cells. BMC Complement. Altern. Med..

[41] Gao J.M., Shen J., Zhang A.L., Zhu W., Zhang X., Liu J.K. (2003). Chemical constituents of the fungus *Leccinum extremiorientale*. Chin. J. Org. Chem..

[42] Kubo M., Ishii R., Ishino Y., Harada K., Matsui N., Akagi M., Kato E., Hosoda S., Fukuyama Y. (2013). Evaluation of constituents of *Piper retrofractum* fruits on neurotrophic activity. J. Nat. Prod..

[43] Bai R., Zhang C.C., Yin X., Wei j., Gao J.M. (2015). Cyathane diterpenoids with neurotrophic activity from cultures of the fungus. J. Nat. Prod..

