# Supplementary material for: Chemical Constituents from Hericium erinaceus Promote Neuronal Survival and Potentiate Neurite Outgrowth via the TrkA/Erk1/2 Pathway
Source: Int J Mol Sci. 2017 Jul 30;18(8):1659. doi: 10.3390/ijms18081659 (PMC5578049; doi:10.3390/ijms18081659)
Supplement: Supplementary file 1 [file ijms-18-01659-s001.pdf]

[Mass Spectrum]
Data : FUKUYAMA-CI.06-May-2016.002 Date : 06-May-2016 15:02
Sample : ZCC-141211(CH4)
Note : MStation
Inlet : Direct Ion Mode : CI+
Spectrum Type : Normal Ion [MF-Linear]
RT : 0.44 min Scan# : 17-k(74)[k=1.0]
BP : m/z 168 Int. : 81.00 (849376)
Output m/z range : 35 to 500 Cut Level : 0.00 %

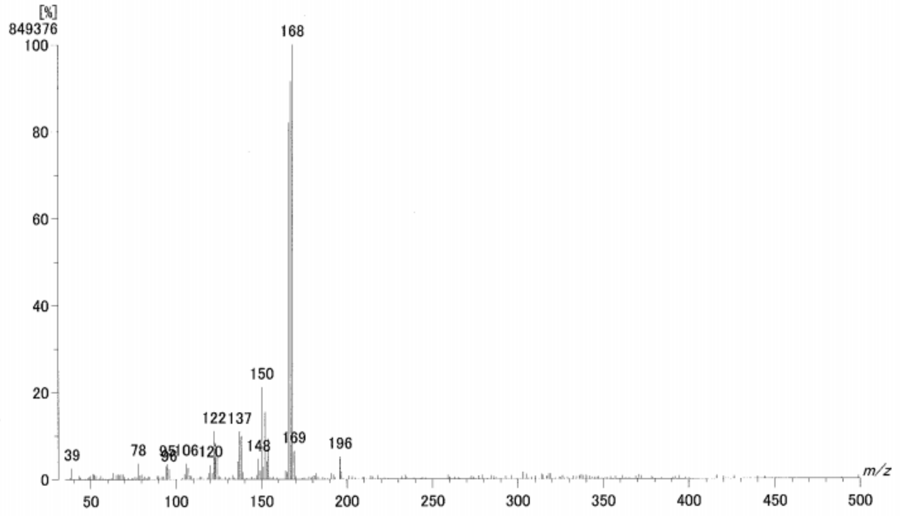

Data : FUKUYAMA-CIHR.26-Jul-2016.001 Date : 26-Jul-2016 10:57
Instrument : MStation
Sample : ZCC-141211
Note : MStation
Inlet : Direct Ion Mode : CI+
RT : 0.40 min Scan# : 9
Elements : C 150/0, H 250/0, N 1/0, O 50/0
Mass Tolerance : 5mmu
Unsaturation (U.S.) : 0.0 – 15.0

	Observed m/z	Int%	Err[ppm / mmu]	U.S.	Composition
1	168.0658	79.68	-1.6 / -0.3	4.5	C8 H10 N O3
2			+14.3 / +2.4	0.0	C5 H12 O6

Figure S1. CIMS and HRCIMS spectra of compound **6** in $CDCl_3$.

ピーク検出 - Memory-2

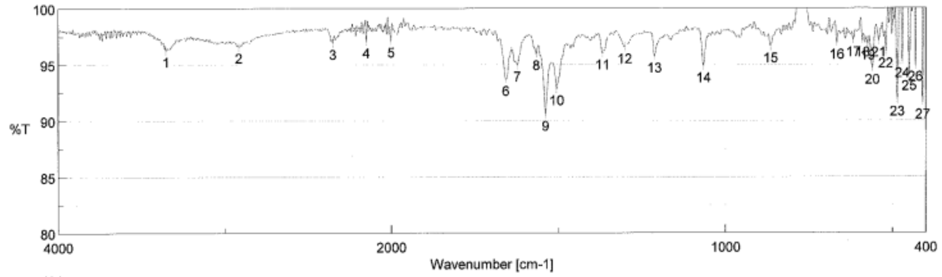

[コメント情報]

試料名	zcc-141211
コメント	
測定者	
所属	
会社	徳島文理大学

[ピーク検出結果]

No.	位置	強度	No.	位置	強度	No.	位置	強度	No.	位置	強度
1	3353.6	96.2653	2	2917.8	96.5977	3	2355.6	96.9275	4	2153.1	97.0458
5	2004.6	97.112	6	1659.4	93.6755	7	1625.7	94.9621	8	1568.8	95.9563
9	1540.8	90.5541	10	1507.1	92.8416	11	1370.2	96.0154	12	1303.6	96.5504
13	1214.9	95.726	14	1067.4	94.8638	15	865.9	96.5962	16	667.3	96.9174
17	618.1	97.1439	18	591.1	97.1136	19	575.6	96.8484	20	561.2	94.6777
21	541.9	97.1052	22	520.7	96.1413	23	486.0	91.8589	24	470.5	95.2604
25	450.3	94.1217	26	431.0	94.9397	27	409.8	91.597			

Figure S2. IR spectra of compound **6** in $CDCl_3$.

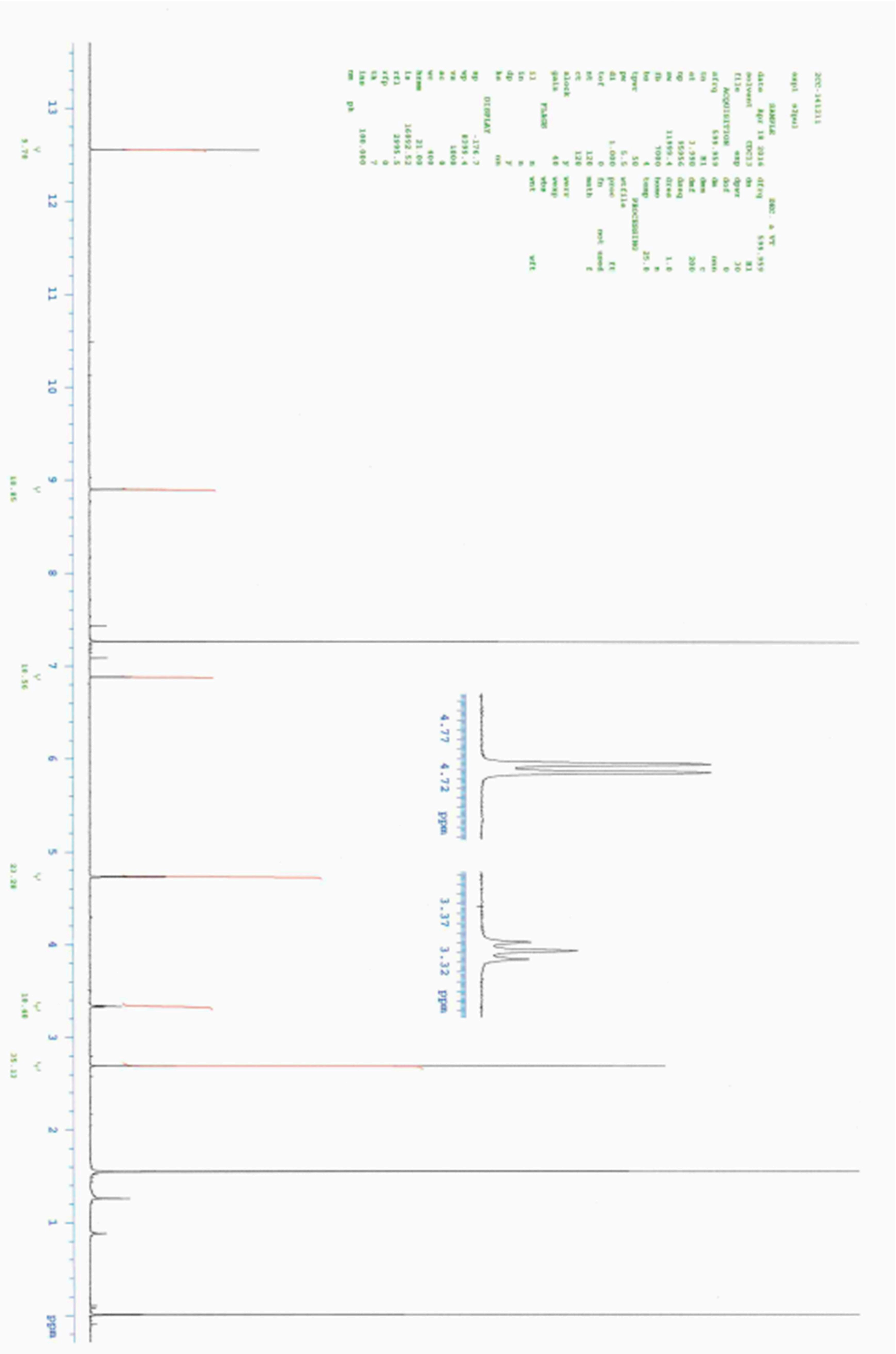

Figure S3. ^{1}H spectra of compound **6** in $CDCl_3$.

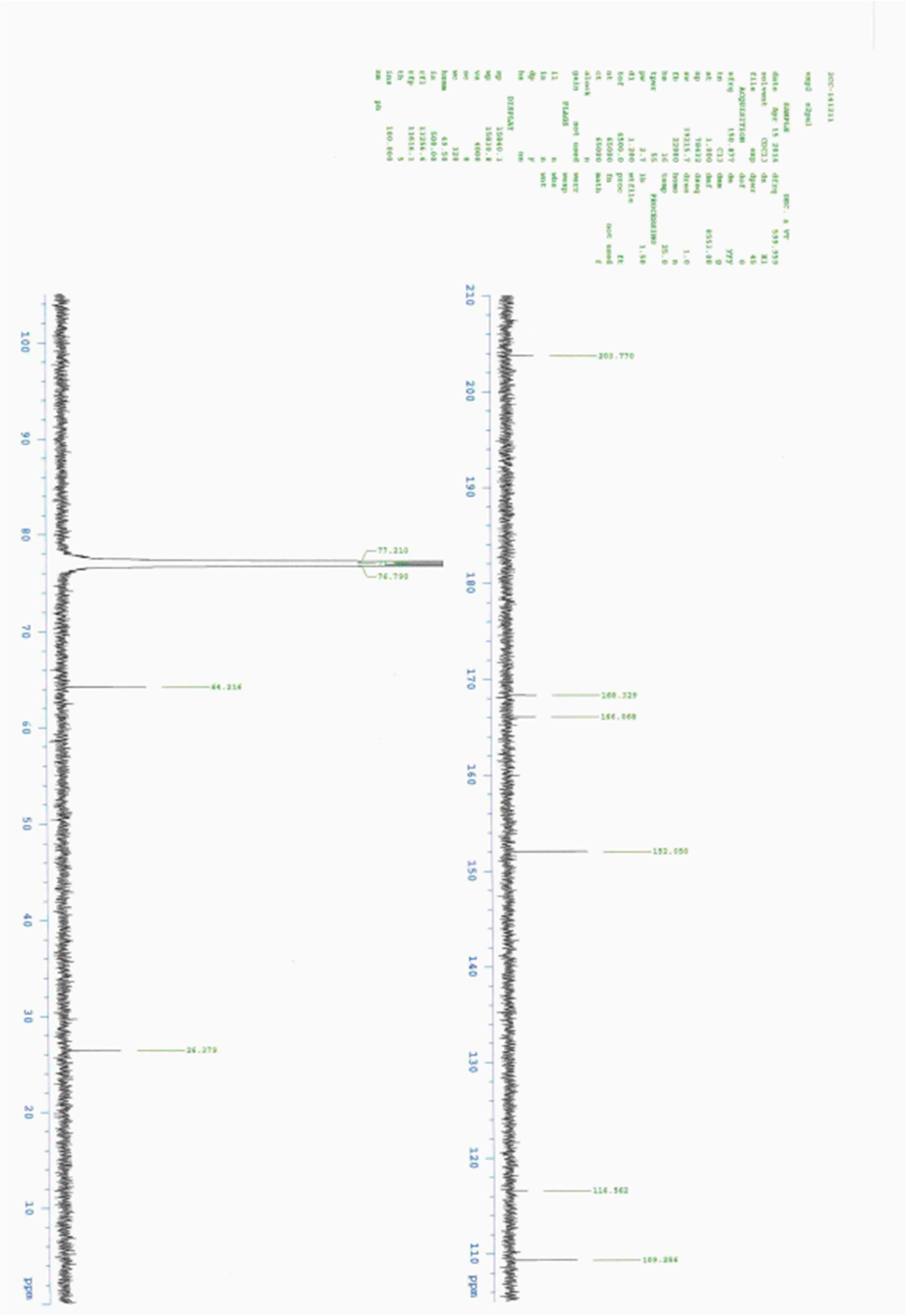

Figure S4. ^{13}C spectra of compound **6** in $CDCl_3$.

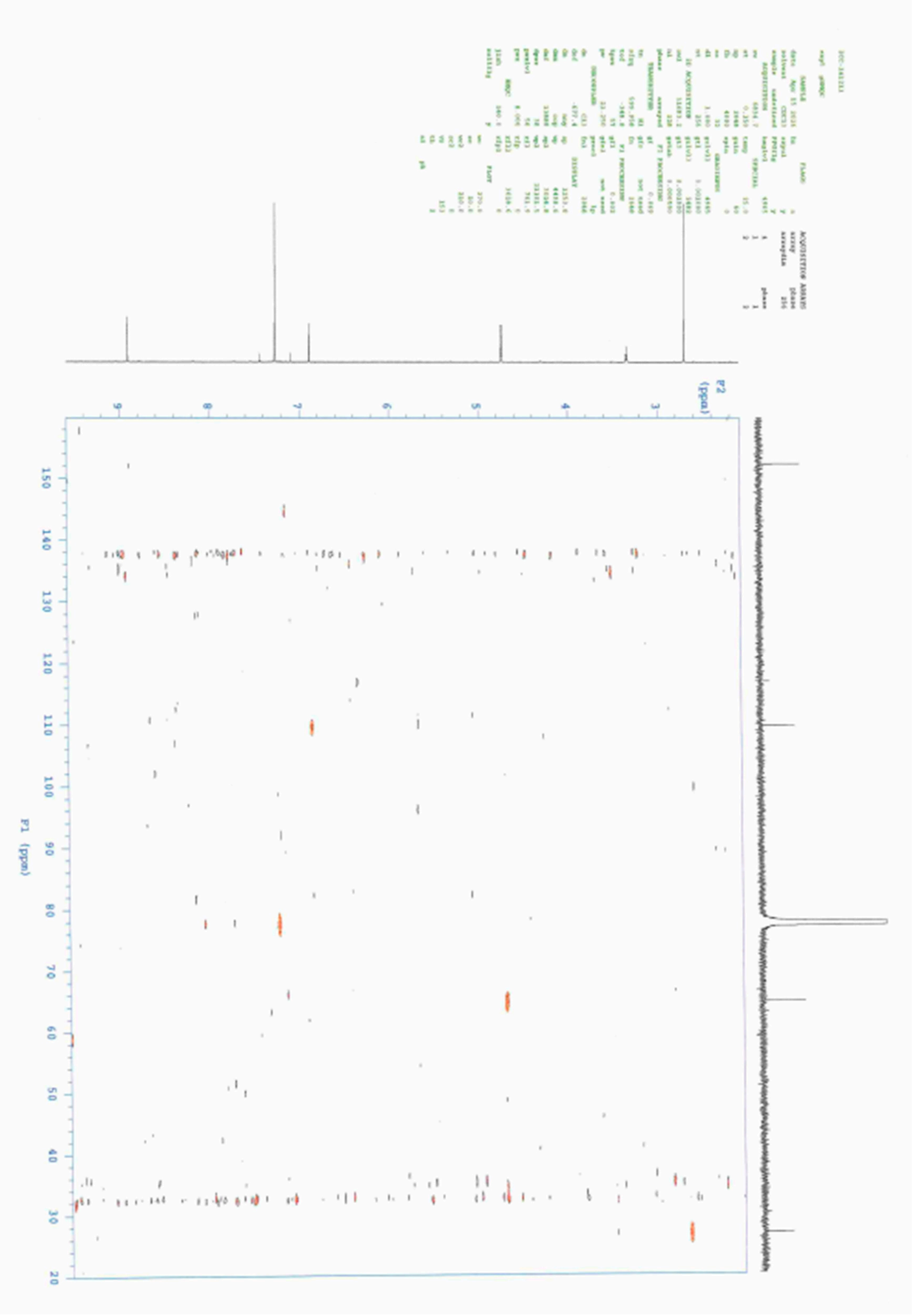

Figure S5. HSQC spectra of compound **6** in $CDCl_3$.

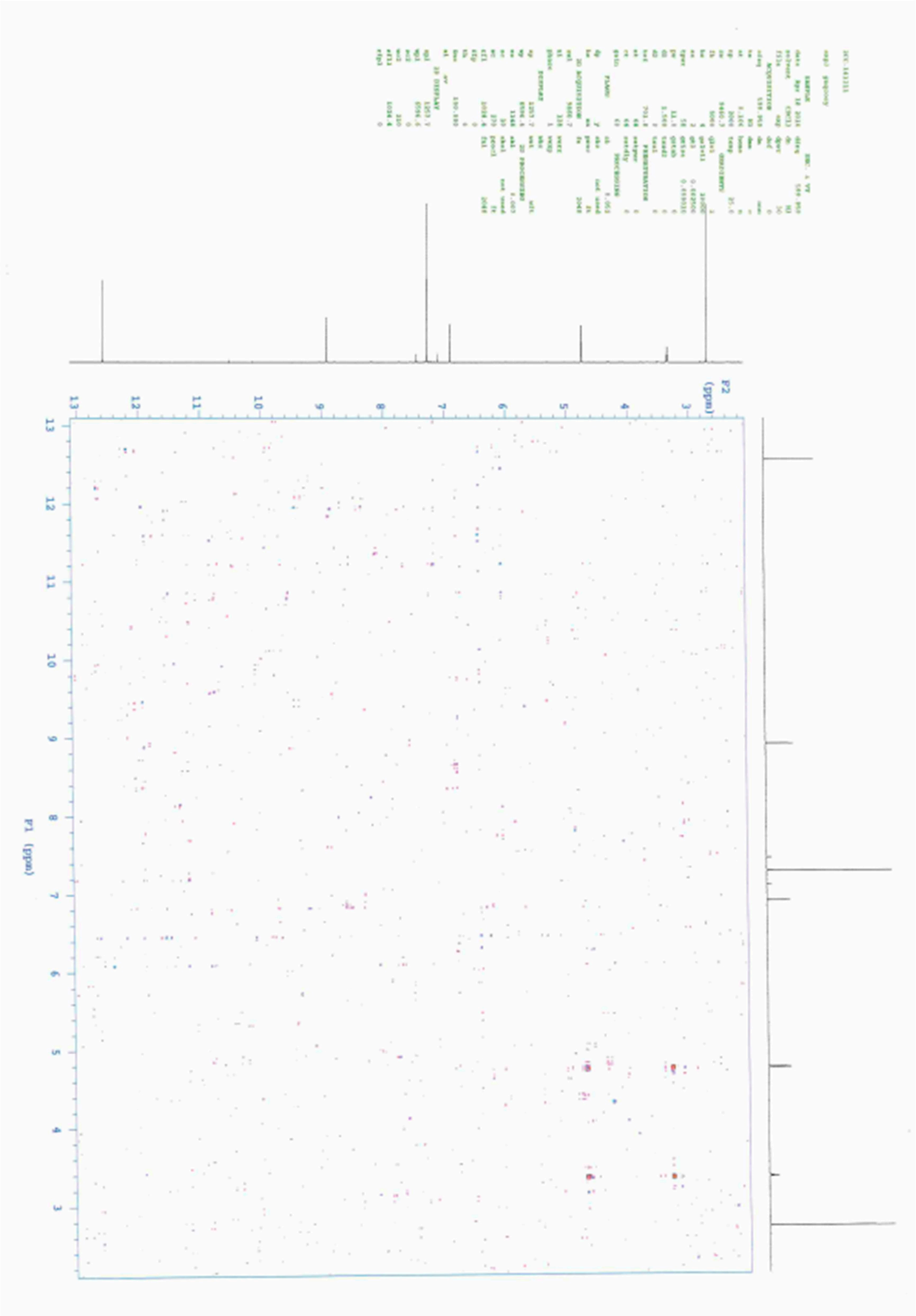

Figure S6. COSY spectra of compound **6** in $CDCl_3$.

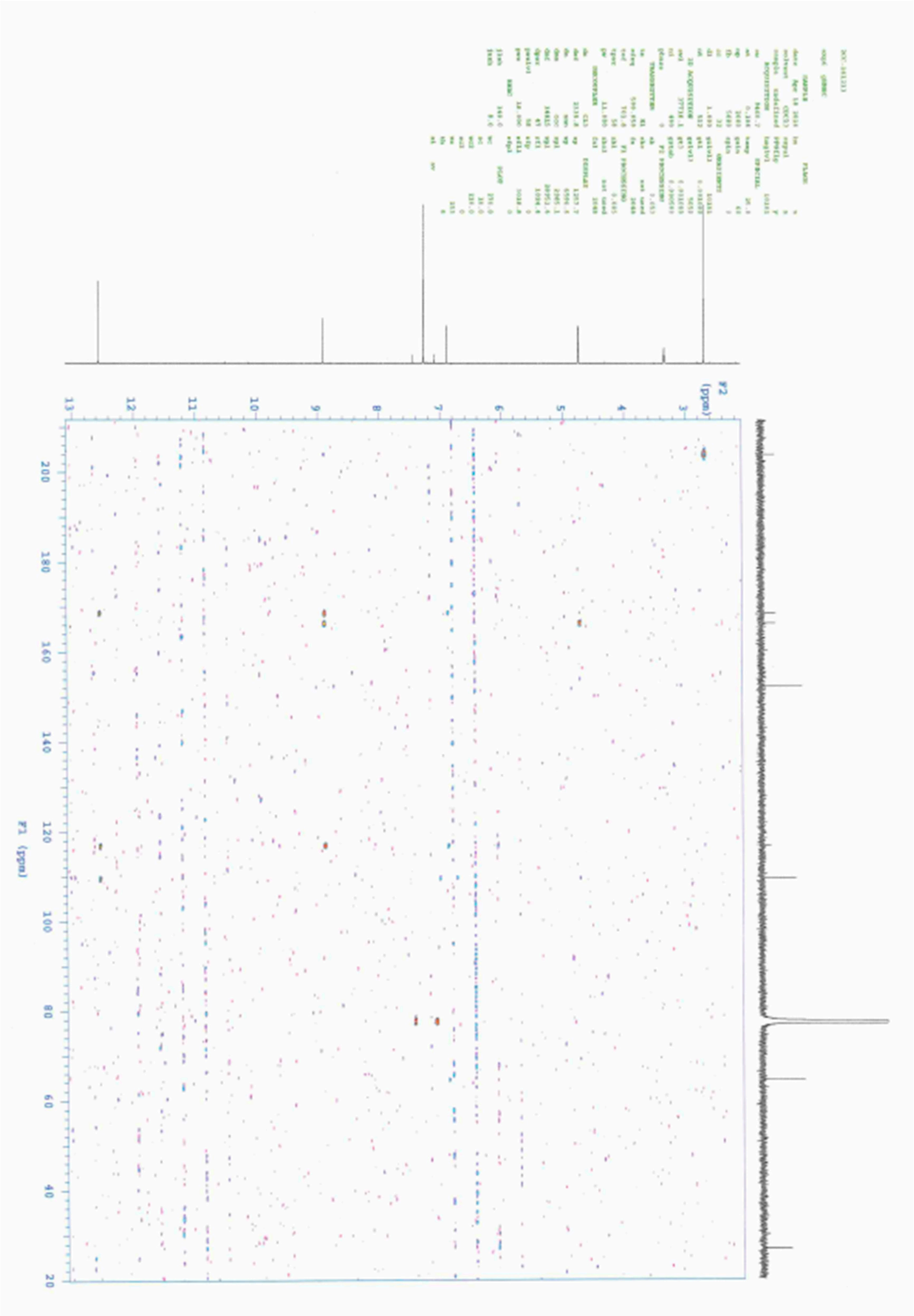

Figure S7. HMBC spectra of compound **6** in $CDCl_3$.

NGF: 0 2ng/mL

The extract: 0 0 12.5 μg/mL 200 μg/mL

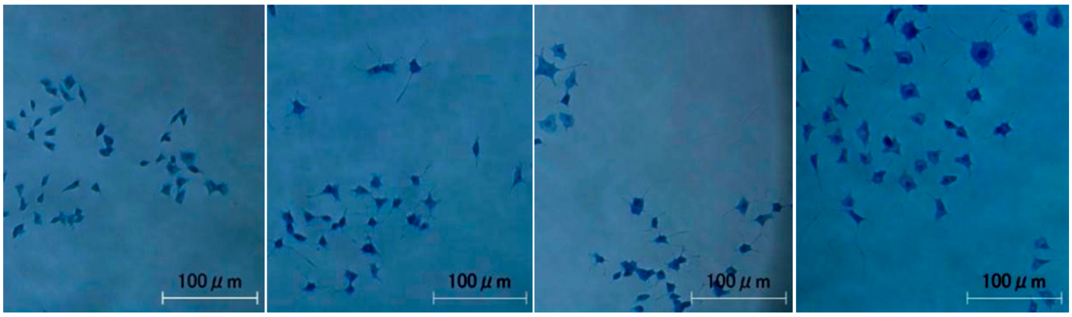

(a)

(b)

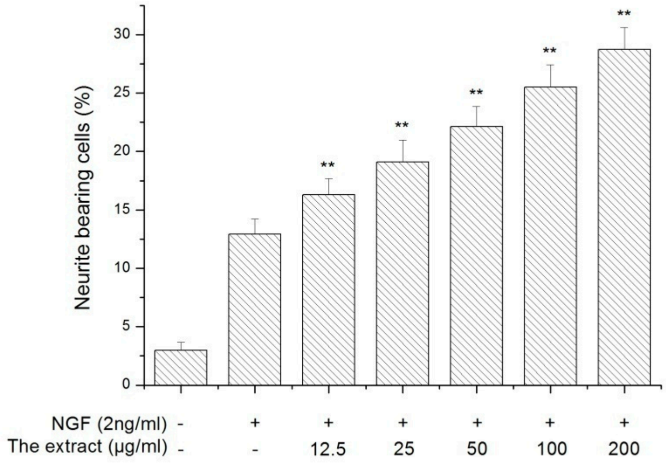

Figure S8. Effect of MeOH extract from the mycelium of *H. erinaceus* on promoting neurite outgrowth in NGF-induced PC12 cells. PC12 cells were seeded in collagen-coated 24-well plates in normal serum medium for 24 h, then changed to low serum medium (2% HS and 1% FBS) and exposure to vehicle (0.1% DMSO) as a negative control, NGF (2 ng/mL) as a positive control, or NGF (2 ng/mL) + the extract (12.5–200 μg/mL) for another 96 h. Scale bar: 100 μM. (**a**) Cell morphology was observed and photographed as described in the analysis of neurite outgrowth. (**b**) Neurite-bearing cells were measured as described in thre analysis of neurite outgrowth. Data are expressed as the mean ± SD from three independent experiments. $^{**}p < 0.01$ represents significant differences compared with NGF-treated PC12 cells (positive control) (ANOVA followed by Dunnett's test).

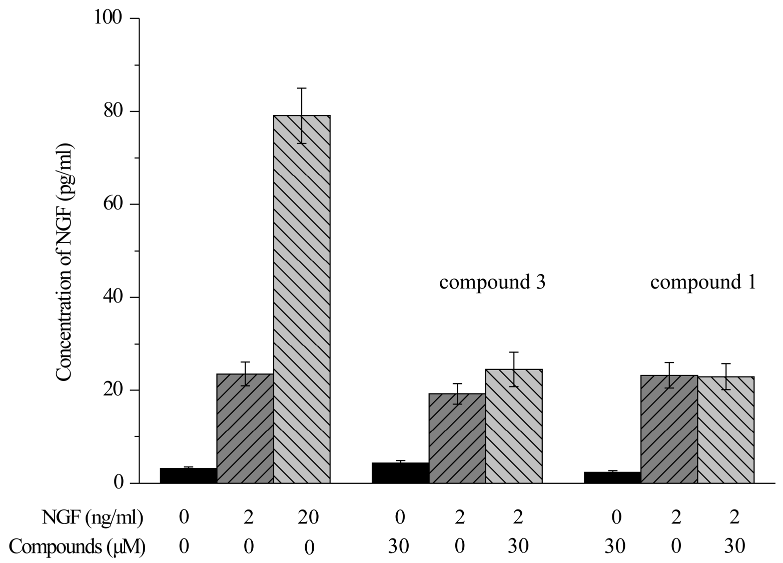

Figure S9. Effects of compounds **1** and **3** on the stimulation of NGF secretion in PC12 cells. PC12 cells were incubated with 30 μM compounds with or without NGF (2 ng/mL) for 96 h and then the NGF level in the conditioned medium was measured by NGF beta rat ELISA Kit (Thermo Fisher Scientific Inc.: Waltham, MS, USA) following the manufacturer's instructions. Recombinant rat beta-NGF was used as standards.
